# Genome-wide Identification, Classification, and Expression Pattern of Homeobox Gene Family in *Brassica rapa* under Various Stresses

**DOI:** 10.1038/s41598-018-34448-x

**Published:** 2018-11-02

**Authors:** Nadeem Khan, Chun-mei Hu, Waleed Amjad Khan, Wenli Wang, Han Ke, Dong Huijie, Zhang Zhishuo, Xilin Hou

**Affiliations:** 1 State Key Laboratory of Crop Genetics and Germplasm Enhancement, Ministry of Science and Technology/College of Horticulture, Nanjing Agricultural University, Nanjing, 210095 P. R. China; 2 New Rural Research Institute in Lianyungang, Nanjing Agricultural University, Nanjing, P. R. China

## Abstract

Homeobox (HB) genes are crucial for plant growth and development processes. They encode transcription factors and responses to various stresses, as reported by recent emerging evidence. In this study, a total of 113 *BraHB* genes were identified in *Brassica rapa*. On the basis of domain organization and phylogenetic analysis, the BraHBs were grouped into nine subclasses, in which homeobox leucine-zipper (HB LZP-III) showed the highest number of genes (28) compared to other subclasses. The BraHBs exhibited similarities in exon–intron organization and motif composition among the members of the same subclasses. The analysis revealed that HB-Knotted was more preferentially retained than any other subclass of BraHB. Furthermore, we evaluated the impact of whole-genome triplication on the evolution of BraHBs. In order to analyze the subgenomes of *B*. *rapa*, we identified 39 paralogous pairs for which synonymous substitution values were lower than 1.00 for further purifying selection. Finally, the expression patterns of BraHBs across six tissues expressed dynamic variations combined with their responses against multiple stresses. The current study provides brief information on the homeobox gene family in *B*. *rapa*. Our findings can serve as a reference for further functional analysis of BraHBs.

## Introduction

Homeobox genes are known to play important roles in the body plan specification of relatively higher organisms at earlier stages of embryogenesis. Plant homeobox genes have been implicated in various processes, such as embryo patterning, development of root, shoot, and floral meristems, vascular development, and various stress responses^1–6^. These genes are considered to be key regulators of plant morphogenesis. Homeobox genes encode a lengthy conserve domain consisting of 60 DNA-binding amino acids, known as homeodomain (HD). Homeobox genes were first isolated in fruit fly (*Drosophila melanogaster*) and were subsequently found to be involved in many aspects of development^7,8^. The characteristic three-dimensional structure of HD comprises three alpha-helices, of which the second and third form a helix-turn-helix DNA-binding motif^9–11^. On the basis of their sequence similarity of homeodomains and co-domain characteristics, BraHB proteins have been classified into seven groups: KNOX, BEL, ZM-HOX, HAT1, HAT2, ATHB8, and GL2^12^. KNOX and BEL belong to the TALE superclass^13^. HAT1, HAT2, ATHB8, and GL2 genes are all characterized by a leucine-zipper motif downstream of the homeodomain^14^ and have been successfully renamed as HD-ZIP I, HD-ZIP II, HD-ZIP III, and HD-ZIP IV, respectively^12,15^, although an alternative classification of these genes into five groups was also proposed (HD-ZIP, GLABRA, KNOTTED, PHD, and BEL)^2^. Furthermore, a comprehensive study on plant homeobox genes was conducted that categorized them into 14 classes, including some new classes, such as NDX, DDT, PHD, LD, SAWADEE, and PINTOX^16^.

Members of the plant homeobox gene family are involved in several development processes. The majority of HD-ZIP I proteins are involved in the regulation of cotyledon development, leaf cell fate determination, and blue light signaling^17,18^. HD-ZIP II proteins participate in shade avoidance responses^19^. Some members of HD-ZIP III modulate the apical meristem formation, vascular development, and maintenance of adaxial or abaxial polarity of leaves and embryos^20^. HD-ZIP IV proteins stimulate the outer cell layer formation of plant organs and monitor anthocyanin pigmentation and epidermal layer maintenance^21,22^. KNOX family members provide support to apical meristem shoot growth maintenance and engage leaf form diversity^23^. They have been also reported to interact with BEL family members for the regulation of hormone homeostasis^3^. WUSCHEL (WOX) family members in *A*. *thaliana* mark cell fate during early embryonic patterning, while some members are also involved in stem cell maintenance and organogenesis^24,25^. These proteins may also be involved in cell differentiation during anther development^26^. Meanwhile, ZF-HD family members are involved with floral development processes in *A*. *thaliana*^27^. The above research emphasized exploring the homeobox proteins and their complex nature.

Chinese cabbage (*Brassica rapa* L. ssp. *pekinensis*) is an important Asian vegetable that is widely cultivated in China, Korea, and Japan^28,29^. The Chinese cabbage genome (Chifu-401-42) was sequenced and assembled recently^30^. It is known to play an important role in global agriculture and horticulture, and exhibits a close relationship with the model plant *A*. *thaliana*. Moreover, it experienced a whole-genome triplication (WGT) event since its divergence from *A*. *thaliana* about 13 to 17 million years ago (MYA)^31^. It also serves as an excellent model system for the study of genome evolution. The genome of Chinese cabbage is based on three subgenomes: least fractionated (LF), medium fractionated (MF1), and most fractionated (MF2). Intriguingly, at the gene density and expression levels, LF is dominant over the other two subgenomes^30,32^. The availability of genome databases for Chinese cabbage, rice, and Arabidopsis has enabled us to search the comparative genomics of the homeobox transcription factor gene family. Characterization of homeobox genes in *B*. *rapa* will be focused in such a way as to provide information about the molecular changes that occur in mechanisms against various stresses such as cold, heat, salt, drought, ABA, and GA. Subsequently, gene modification can proceed further by using resistant *Brassica* varieties.

In this study, we identified 113 homeobox genes in Chinese cabbage based on their genome sequences and categorized them into nine subclasses. Gene duplications and chromosomal locations were also investigated to support our findings. Expression profiles under six different stress treatments (ABA, GA, drought, salt, heat, and cold) were evaluated to determine the responses of *BraHB* genes in Chinese cabbage (Chifu-401-42). Moreover, cis-element promoter functions were also predicted. Our results provide novel insights into the stress responses of *BraHB* genes and convey a clear image to understand the construction and function of homeobox genes in Chinese cabbage.

## Results

### Identification, Classification, and Comparative Analysis of BraHBs in *B*. *rapa*

**Figure 1:**
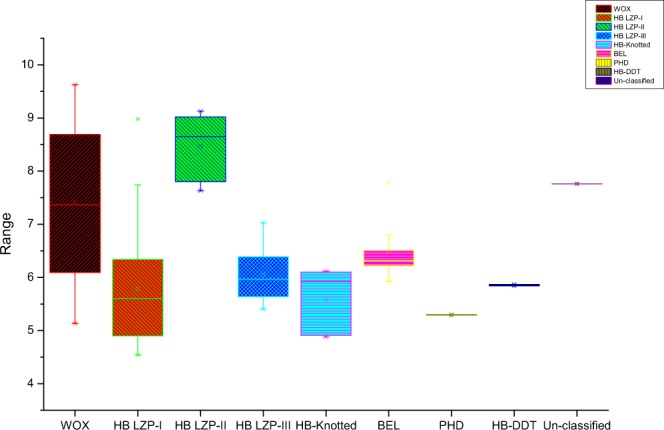
Indication of isoelectric point (pI) values among different subclasses of BraHB.

**Figure 2:**
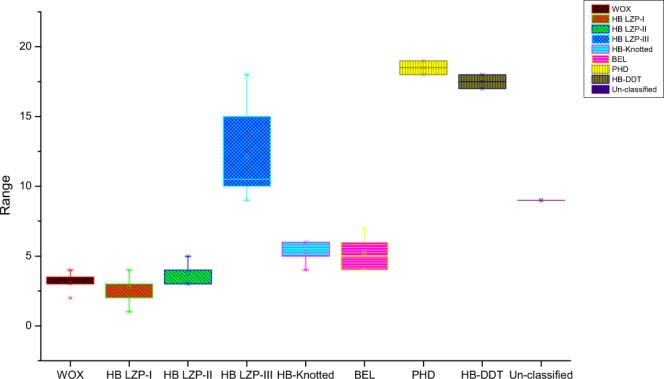
Number of exon among subclasses of BraHB.

**Figure 3:**
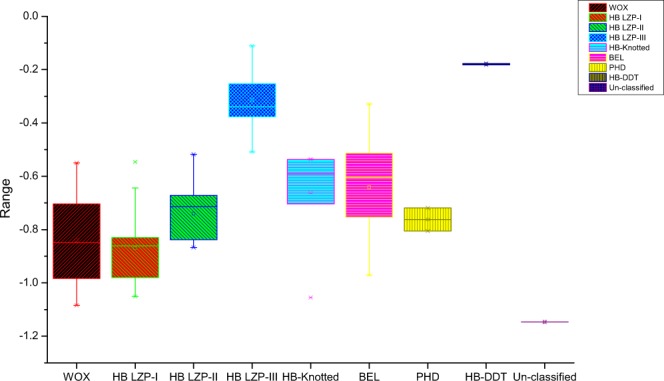
Grand average of hydropathicity (GRAVY) among the subclasses of BraHB.

**Figure 4:**
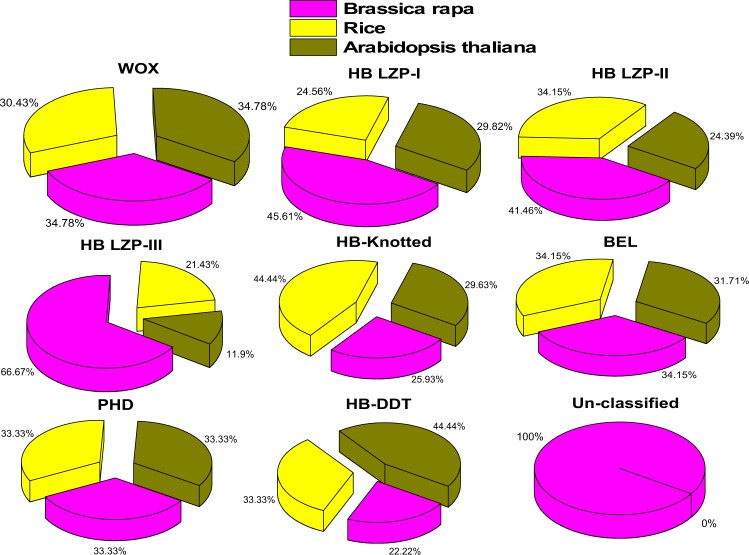
Relative classification patterns of genes among three species.

Based on our putative *B*. *rapa* genome studies^33^, we used sequences of rice and *A*. *thaliana* as queries and hidden Markov model (HMM) profile to confirm the HD in the *B*. *rapa* genome. According to the HDs, we identified 113 BraHB proteins (Table 1). The distribution pattern of homeobox in *B*. *rapa* was divided into nine subclasses, including two major classes, HD ZIP (HD ZIP-I, HD ZIP-II, HD ZIP-III) and TALE (KNOX and BEL), and three subcategorizations of WOX, HB-DDT, and PHD based on previous reports in plants^16^. The complete information related to BraHBs is listed in Supplementary Table S1, including gene identifier, protein length, cDNA length, genomic location, and subcellular prediction. The theoretical isoelectric point (pI) for all BraHB ranges from 4.54 to 9.63, with a median of 6.56, is shown in Fig. 1. The data of the number of exons (Fig. 2) show complex ranges for different subclasses, but the normal range was calculated as 1–19, with a median of 6.33. The subcellular predictions for all the BraHBs (Supplementary Table S1) show that the majority of proteins were localized in the nucleus, cytoplasm, and mitochondria, although some proteins were located in different organelles, i.e., Golgi body, plasma membrane, chloroplast, and others. In order to further characterize the BraHBs, we analyzed the physicochemical properties of the putative proteins (Supplementary Table S1). The value for the grand average of hydropathicity ranges from -1.147 to -0.111, which appeared to be negative and represent hydrophilic behavior (Fig. 3). The 113 members of BraHB were designated as BraHB 1 to BraHB 113 (Supplementary Table S1). In this study, the largest grouping of proteins was identified in subclass HB LZP-III (28 proteins), followed by HB LZP-I (26), with HB ZIP proven as the major class of the homeobox family. The lowest number of proteins was observed in PHD and HB-DDT, with two each. So the expansion pattern of homeobox in the *B*. *rapa* genome was similar to that of rice and *A*. *thaliana*, i.e., the number of genes was 113. We also analyzed the relative shares of different subclasses of BraHBs based on the number of genes in them (Fig. 4). Consistently, HB LZP-III showed the highest number of genes in *B*. *rapa* (66.77%) compared to the other two species, while the PHD subclass shared the same number of genes (33.33%).

**Table 1:** Homeobox identified genes of *Brassica rapa* classified based on their domain and phylogenetic relationships.

Subclasses	Identified Genes
WOX	16
HB LZP-I	26
HB LZP-II	17
HB LZP-III	28
HB-Knotted	7
BEL	14
PHD	2
HB-DDT	2
Unclassified	1
**Total**	**113**

### Expansion Analysis and Characteristic Structure of BraHBs

**Figure 5:**
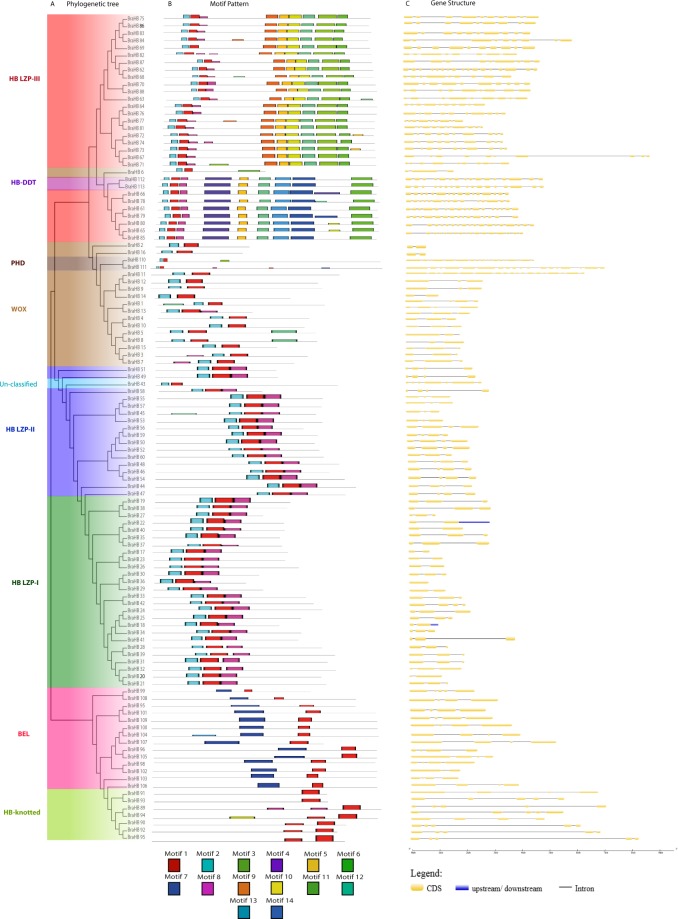
Phylogenetic tree, protein motif, and gene structure of BraHBs. (**A**) The phylogenetic tree was constructed by MEGA 7 using the maximum likelihood method (1000 bootstrap). (**B**) The conserved motifs of BraHBs were elucidated by Multiple EM for Motif Elicitation (MEME). Different motifs and their positions are represented by different colors, numbered 1–14 at the bottom. (**C**) The exon–intron and upstream/downstream regions are represented by yellow boxes, gray lines, and blue box, respectively. At the bottom of the figure the relative position is proportionally displayed based on the kilobase scale.

To examine the expansion and evolutionary relationships among different subclasses of BraHBs, a phylogenetic tree was constructed using MEGA 7.0 software. The maximum likelihood method with 1000 bootstrap replications was used (Fig. 5A). The phylogenetic results showed that the distribution of BraHBs was subcategorized into further divisions: HB ZIP contained three subclasses with different members of BraHB (HB ZIP-I (26), HB LZP-II (17), and HB LZP-III (28), HB-Knotted (7) and BEL (14)). WOX (16), HB-DDT (2), PHD (2), and uncharacterized (1) proteins were identified, which were consistent with reports on *A*. *thaliana* and rice^33^. Interestingly, all of the subclasses of BraHBs were grouped together in a phylogenetic tree except WOX, which was considered to be diverse in nature. To better understand motif composition in BraHBs, all of the putative protein sequences were analyzed by Multiple EM for Motif Elicitation (MEME) software (version 4.12) for the conserved motifs^34^. In total, 14 motifs were identified, designated as motifs 1 to 14, by calibrating their width ranges from 12–100 (Fig. 5B). At the same time, the LOGO of BraHB proteins was also procured with the help of MEME (Supplementary Figure S1). The number of conserved motifs varied in the range of 2–10 in all proteins. We noticed that motif 1 was the most dominant, as it was found in all BraHBs, followed by motif 2. The other motifs were specific to one or two subclasses; for example, motifs 5, 6, 9, and 12 were specific to HB LZP-I, whereas motifs 1 and 2 were specified to WOX, which might be due to the functional similarities within subfamilies. The varied nature of different motifs within BraHBs suggests inconsistency in their functions. The exon–intron organization was employed using Gene Structure Display Server (GSDS) 2.0 software, in order to gain additional information with respect to conservation and diversification of BraHBs, as the gene structure is more closely related to the function of the gene and, together with phylogenetic tree construction, it reflects the close relationship of different subclasses of BraHBs (Fig. 5C). Most of the BraHBs within the same subclasses exhibited similar patterns of structure with respect to the same number of exons and introns, ranging from 4–19 and 2–17. On the other hand, a majority of the genes showed similar gene structure; however, significant diversification was also detected among BraHB subfamilies. We also calculated the genetic distance to estimate the relationships among the nine subclasses using box plots (Supplementary Figure S2a–c). Notably, the genetic distance between BEL and HB-Knotted was shorter than the others (Supplementary Figure S2a). Most of the groups show close distance except HB-LZP III vs. HB-DDT (Supplementary Figure S2b). Also, most of the groups show similarity while belonging to different subclasses of BraHBs (Supplementary Figure S2c). The similar patterns among subclasses suggest that most of them share a common evolutionary origin.

### Different Retention of BraHB Genes Following the Whole Genome Triplication Event

**Figure 6:**
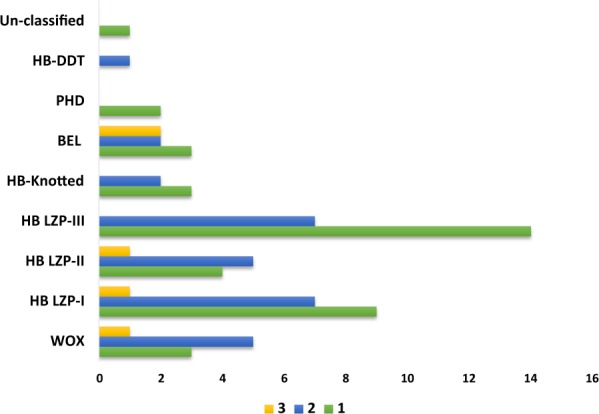
Copy number of variation and gene retention in different protein groupings.

**Figure 7:**
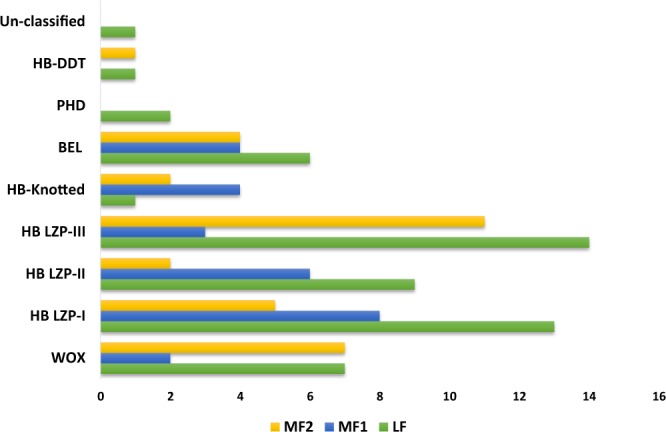
Representation of number of genes between the subclasses of BraHB within the three subgenomes of *B*. *rapa*.

**Figure 8:**
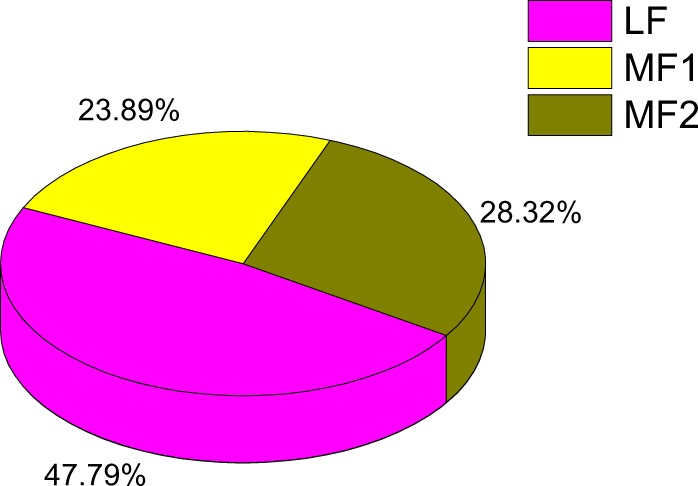
Relative percent of the number of genes within three subgenomes of *B*. *rapa*.

In order to explore the influence of WGT on the evolution of BraHBs, we studied the gene retention of BraHB after WGT. The *B*. *rapa* genome contains ∼42,000 genes after its divergence from the *A*. *thaliana* genome, which was consistent with ∼30,000 genes. For this occurrence, a considerable number of genes were lost after triploidization in *B*. *rapa*^30^. The results of different retention of genes in the syntenic region with respect to subclasses were demonstrated as follows: WOX (16/20), HB LZP-I (26/31), HB LZP-II (17/19), HB LZP-III (28/36), HB-Knotted (7/7), BEL (14/19), PHD (2/4), HB-DDT (2/2), and unclassified (1/1) (Supplementary Table S2). In order to compare the different retention of genes among subclasses of BraHBs, we also counted the copy number of genes and analyzed their distribution patterns across the three subgenomes of *B*. *rapa*: least fractionated (LF), medium fractionated (MF1), and most fractionated (MF2). As shown in Fig. 6, the most copies of genes (14) were found in HB LZP-III, followed by HB LZP-I (9), and HB LZP-II (4). We also counted the number of genes in the subgenomes. All nine subclasses of BraHBs showed various numbers of genes. In summary, HB LZP-I and HB LZP-III displayed similar numbers of genes, 26 and 28, respectively. Additionally, both of them carried a high number of genes, 13 and 14, in the LF genome compared to other subclasses of BraHBs (Fig. 7). Overall, a majority of genes (47.79%) were located in the LF genome, as described in Fig. 8. As for the other subgenomes, MF2 contained 28.32%, while the least number of genes was found in MF1, 23.89%.

### Chromosomal Localization and Synteny Gene Analysis of BraHB

**Figure 9:**
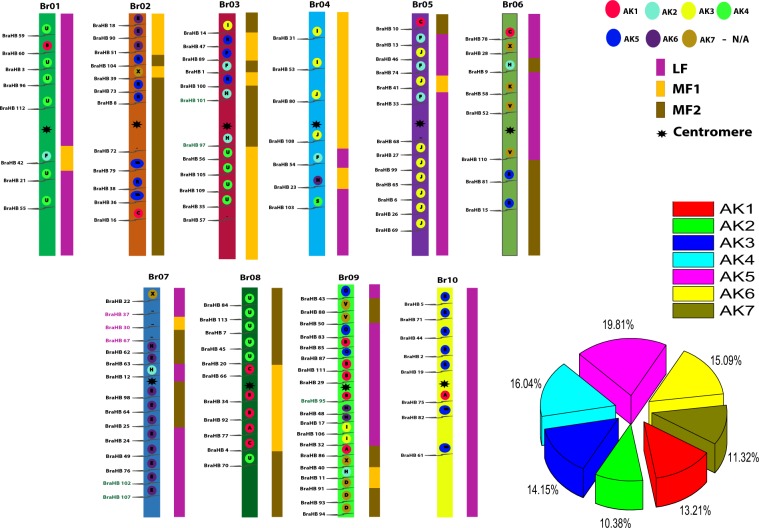
Chromosome locations of BraHBs were obtained from the generic file format (GFF) file and displayed by using MapChart (modified by Adobe Illustrator). The ancestral karyotypes are marked in different colors. Duplication types are marked as follows: segmental in black, tandem array in green, and dispersed in purple.

**Figure 10:**
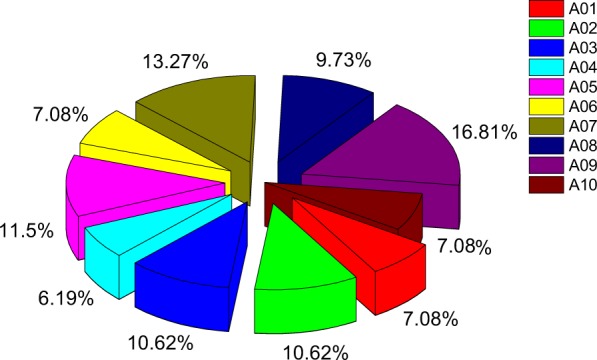
Relative shares of different subclasses of BraHB from A01–A10 chromosomes of *B*. *rapa*.

All *BraHB* genes were mapped to the 10 chromosomes of Chinese cabbage, which were distributed in a random manner (Fig. 9). On every chromosome, the proportion of genes was found to be random. Chromosome A09 contained the most genes (19), whereas chromosome A04 had the least (7). The other chromosomes, A01, A02, A03, A05, A06, A07, A08, and A10, contained 8, 12, 12, 13, 8, 15, 11, and 8 genes, respectively. Meanwhile, according to a previous study^11^, we also demonstrated in *B*. *rapa* genome the 24 conserved ancestral genomic blocks (labeled A–X). The colors were arranged according to the position of these blocks in a proposed ancestral karyotype (AK1–7). We observed that most of the *BraHB* genes clustered together in a region of AK5 (19.81%), followed by AK4 (16.04%), and AK (15.09%), whereas the least amount of genes were located in AK2 (10.38%). Based on shares among subclasses of BraHBs, chromosome A09 had the highest share (16.81%), followed by A07 (13.27%), while A01, A06, and A10 had equal shares of 7.08% (Fig. 10).

**Figure 11:**
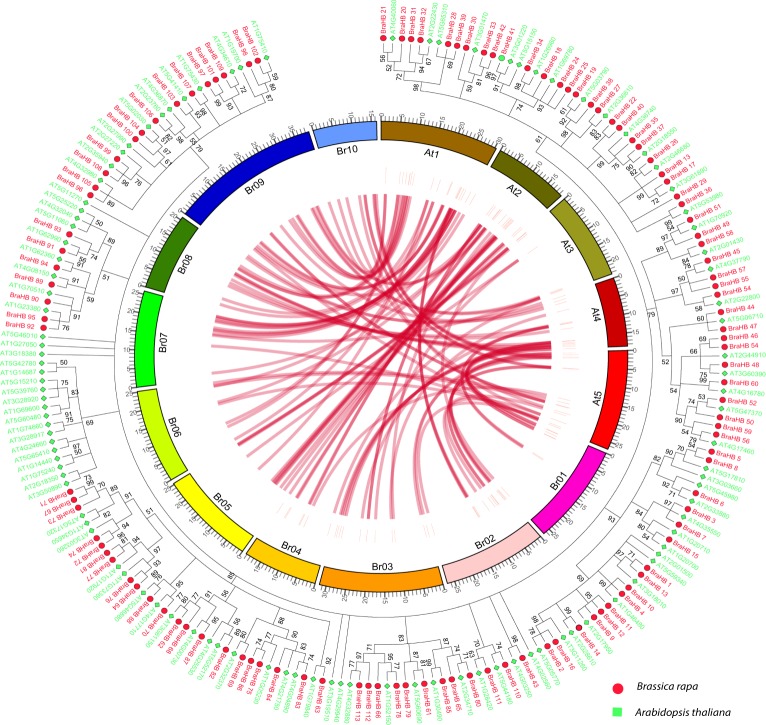
Collinear correlation for all genes of BraHB displayed between *B*. *rapa* and *A*. *thaliana* along with phylogenetic tree. The 10 Chinese cabbage chromosomes (Br01–Br10) and five *A*. *thaliana* chromosomes (At1–At5) are shown in different random colors. The illustration was drawn using Circos software. The phylogenetic tree was constructed by MEGA 7 using the maximum likelihood method (1000 bootstrap).

Concurrently, the types of duplication genes were also identified and classified by using the MCScanX program. In total, we identified five tandem genes that were located on three different chromosomes, of which four were on A03 and A07 (two on each), with single duplication on A09. We also identified three dispersed genes that were only located on chromosome A07. The number of genes between the subgenomes and non-synteny ortholog are also presented (Supplementary Figure S3). The synteny gene relationship between chromosomes of *B*. *rapa* and *A*. *thaliana* was summarized by using Circos software along with the phylogenetic tree (Fig. 11).

### Analysis of Putative Regulatory Cis-Element in BraHBs

**Figure 12:**
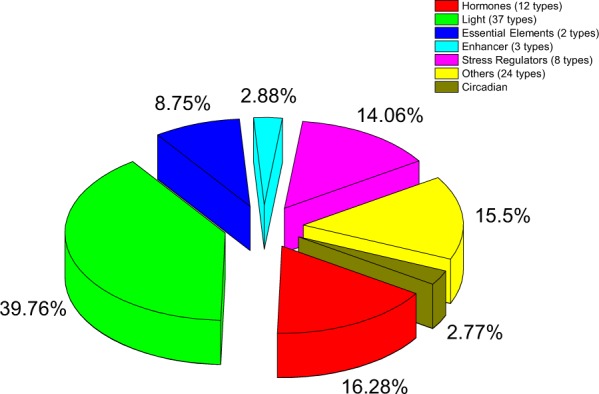
Relative percent of different genes of BraHB according to the distribution patterns of cis-elements.

Transcription factor is one of the key factors involved in the regulation and expression of genes by either promotion or suppression. However, transcription factor is also under the control of other regulators upstream during various biological processes in plants. Therefore, homeobox genes may be assumed to regulate binding of the promoter regions and control the cascade reaction that mainly occur in plants on certain occasions. For this reason, we carried out cis-regulatory element analysis for the identification of transcriptional regulation in the promotor region (2 kb upstream) of BraHBs in *B*. *rapa*. We utilized the PlantCARE database to identify the various cis-elements. Consequently, we figured out a number of different elements involved in various physiological processes of plants. For example, for the processes related to hormonal pathways, we identified 11 types of cis-elements including light (37), essential elements (2), enhancer (3), stress-related (8), and others (25), which are presented in Supplementary Table S3. There were many hormone-related elements, which were found in most of the promoter sequences, i.e., CGTCA, ERE, GARE, ABRE, and AuxRR-core. They are mainly involved in hormone signaling, such as methyl jasmonate, ethylene, gibberellin, abscisic acid, and auxin, suggesting that they may control the regulatory expression of BraHBs. Interestingly, for light regulation factor, we found many different elements (37) that were common in most of the genes of BraHB. We speculated that they may be involved in plant energy metabolism. For stress-related genes, common cis-elements were HSE, MBS, and skn-1-motif, which indicated that they may help in the stress mechanism and other developmental pathways. Other important cis-elements like Box-W1 are responsible for fungal elicitor. Several transcriptional regulation cis-elements, 5UTR Py-rich stretch, CAAT, and TATA, were also found among BraHBs. Figure 12 describes the analysis based on the number of *BraHB* genes involved under various cis-elements. Most of the genes were involved in light-responsive types of promoters (39.76%), followed by hormones (16.28%) and other stress-related genes (15.5%). Notably, the promoter sequences of BraHBs involved in plant circadian cis-elements might be involved in controlling the plant environment, such as periodicity and temperature compensation.

### Expression Pattern Analysis in Different Tissues of BraHBs

**Figure 13:**
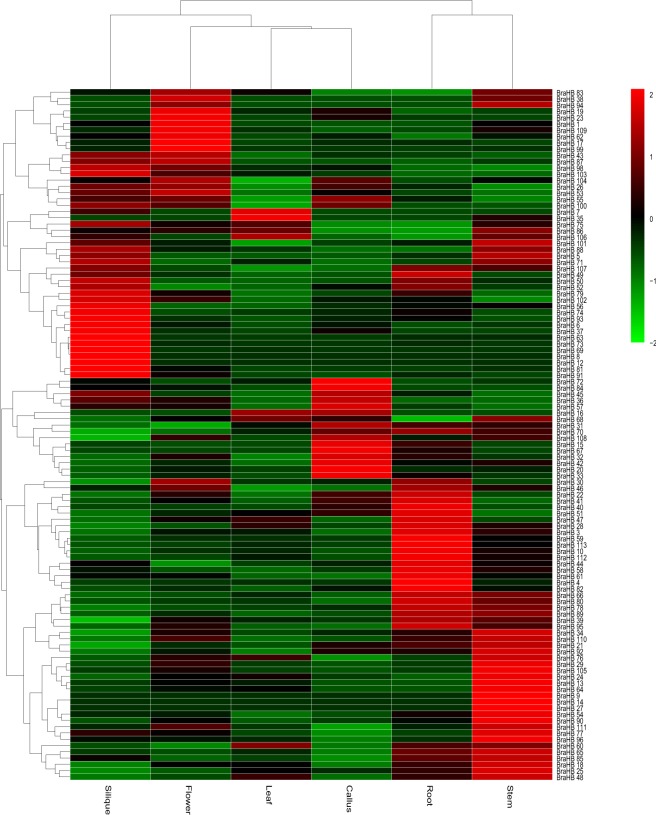
Heatmap of expression profiles (in log2-based fragments per kilobase of transcript per million mapped reads (FPKM)) of BraHBs in six tissues (stem, root, callus, leaf, flower, and silique). The expression levels are exhibited by the color bar.

**Figure 14:**
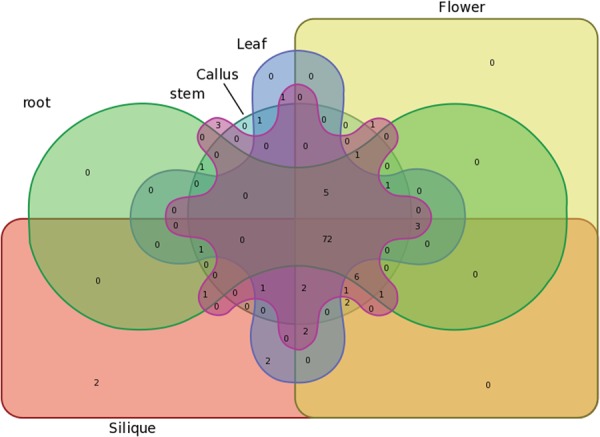
Venn diagram of the tissue expression of BraHBs.

To characterize the expression patterns of BraHBs, we utilized publicly available resource data^35^ for six tissues: root, stem, leaf, flower, silique, and callus. A heatmap for these tissues was generated to display the expression patterns and clustering of *BraHB* genes. Most of the genes highlighted different expression patterns, while some exhibited similar expression (Fig. 13 and Supplementary Table S4). According to the results of expression of *BraHB* genes in different tissues, high expression was recorded in roots (81.4%), followed by stem (88.49%), leaf (80.3%), flower (85.84%), silique (84.95%), and callus (83.18%). Noticeably, our results show approximately higher expression in all the tissues. Among all the *BraHB* genes, the relative expression pattern in stem was higher (88.49%). Overall, the relative expression exhibited a consistent level of about 83.95%, which was expressed across all six tissues of *BraHB* genes. Likewise, clustering of different tissue-specific genes was also demonstrated (Fig. 14). Interestingly, two novel genes were found in silique, which might suggest that these genes have a tissue-specific role.

### Syntenic Paralog Pairs and Prediction of the Evolutionary Fate of BraHB

**Figure 15:**
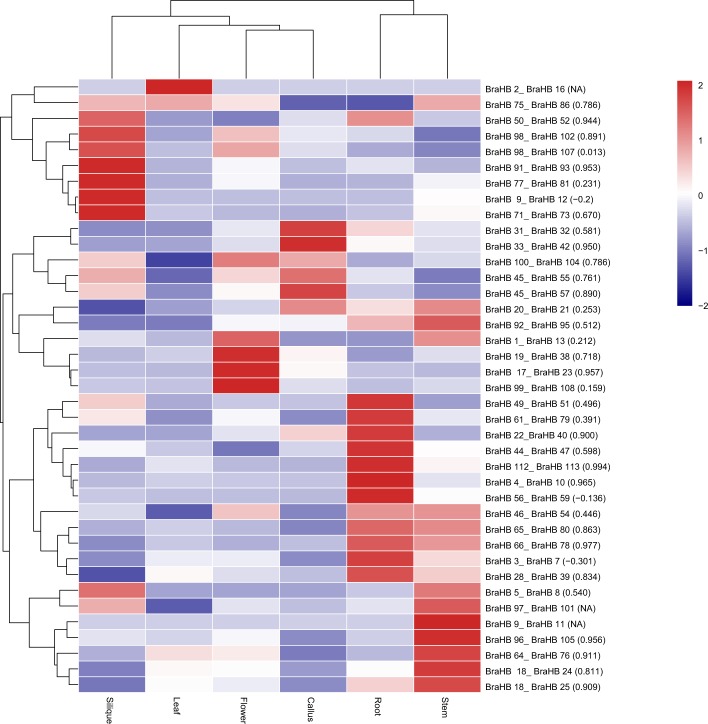
Heatmap of expression profiles for BraHBs 39 paralogous pairs in six tissues: stem, root, callus, leaf, flower, and silique. Pearson correlation coefficients (PCCs) are shown in brackets. NA indicates no available results for PCC.

**Figure 16:**
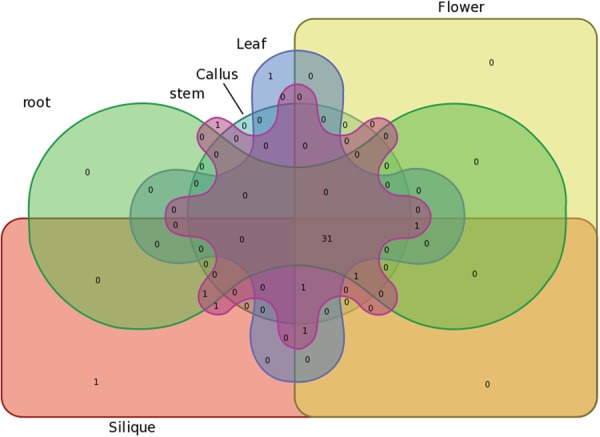
Venn diagram of the tissue expression of BraHBs.

Based on a literature review and outcomes from our study, we made an attempt to use the publicly available resource data of RNA-seq.^35^. The information based on gene expression provided an opportunity to understand the mode and tempo of duplicated genes. We used 39 paralogous pairs of BraHB for gene expression profiling across six tissues (root, stem, leaf, flower, silique, and callus). The results, presented in Fig. 15 and Supplementary Table S5, show high relative expression among all the tissues: root (87.17%), stem (97.43%), leaf (89.74%), flower (89.74%), silique (94.87%), and callus (84.61%). The clustering image of the paralogous pairs of BraHB showed that only one specific gene was found in silique for a tissue-specific role among the six tissues (Fig. 16). Additionally, we also calculated the correlations among the 39 paralog pairs. Based on our results, the Pearson correlation coefficient (PCC) value was >0.6 in about 20 pairs. The higher PCC values between these paralog pairs indicated their close relationship and the involvement of functional conservation or subfunctionalization after the event of duplication. In our results, three pairs were negatively co-related among all the *BraHB* genes, as these paralog pairs may indicate neofunctionalization. Interestingly, two pairs of genes from WOX and BEL showed no expression, because one copy of each did not exhibit any expression pattern. So the PCC values for these paralog pairs were not recorded (NA), which might be due to pseudogenization during the process of evolution.

The three subgenomes of *B*. *rapa* are evolutionary products of WGT and contain many synteny blocks between them. Syntenic paralogs are genes that are located in the syntenic fragments. Syntenic BraHB paralog pairs among LF, MF1, and MF2 were identified by searching “syntenic gene” in the Brassica Database (BRAD). In total, we also observed 39 pairs of paralogous genes. Synonymous (*Ks*) and nonsynonymous (*Ka*) values were calculated (Supplementary Table S6) to monitor the selective pressure on these paralog pairs. Interestingly, we found that the ω = *Ka/Ks* ratios of 39 syntenic paralogs were estimated below 1.00, therefore they may lie in the purifying selection. In addition, the duplication time of these paralogs pairs was calculated by using a relative *Ks* measure as a proxy for time. The highest estimated duplication time of BraHB paralogous pairs was calculated as 16.87 MYA, with an average of 10.63 MYA. Our results suggest that stronger selective pressure on BraHBs forces them to duplicate earlier for their survival and leads to the speculation of BraHBs with varied and complex function.

### Differential Expression of BraHBs in Response to Abiotic Factors and Their Correlation

**Figure 17:**
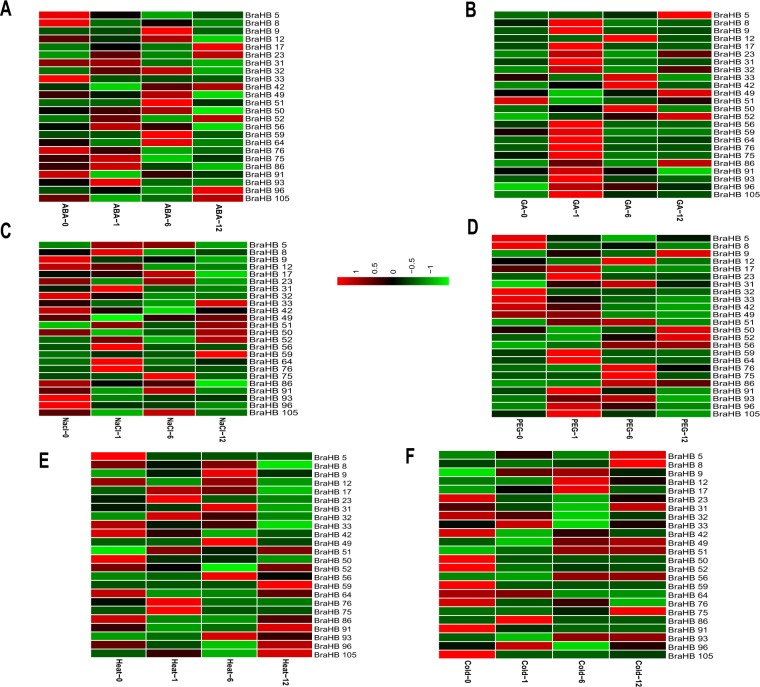
Expression analysis of *BraHB* genes under six abiotic stress treatments in *B*. *rapa* (**A–F**). Heatmap representation the *BraHB* genes under six stress treatments: ABA, GA, NaCl, PEG, heat, and cold.

**Figure 18:**
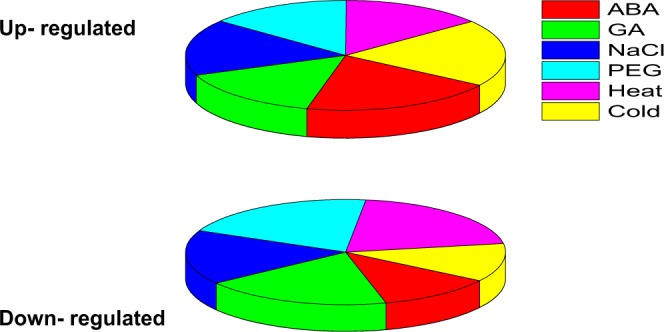
Expression patterns of *BraHB* genes in response to multiple treatments.

**Figure 19:**
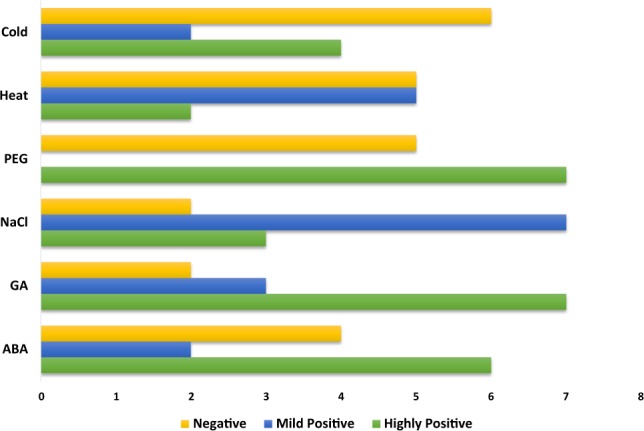
Pearson correlation coefficient values among the paralogous pairs of genes.

Plant growth and development can be adversely affected by the uncertainty of climate change, such as fluctuations in temperature, salinity, and drought conditions that may limit crop productivity^36–38^. Since homeobox genes are known for their important role in the regulation of gene expression patterns under various abiotic stress factors, we selected 12 paralogous pairs of genes and analyzed them under multiple treatments: ABA, GA, PEG, NaCl, heat, and cold (Fig. 17 and Supplementary Table S7). A range of different expression profiles of selected pairs of BraHBs was observed as a result of these stresses. In general, in the case of ABA and cold stresses, the results of qRT-PCR showed that about 73% of genes were upregulated and 27% were downregulated (Fig. 18) on the basis of their different levels of treatment, i.e., 0, 1, 6, and 12 h. For GA, there was a slight decrease, with about 56% of genes upregulated and 44% downregulated, while NaCl had a slight increase, with 61% upregulated and 39% downregulated genes. Intriguingly, the drought and heat treatments show identical results, with 53% upregulated and 47% downregulated genes. For better understanding, a correlation was made among 12 paralogous gene pairs with respect to multiple treatments and an estimation of PCC was done. The correlation was designated as highly positive, mildly positive, or negative on the basis of PCC values^39^: >0.6, highly positive; 0.5–0, mildly positive, and <0, negative. For both GA and PEG treatments, seven pairs of paralogs showed high correlation (PCC > 0.6), followed by ABA with six pairs, while the most negative correlation was observed with cold stress (Fig. 19 and Supplementary Table S8). Moreover, these correlations were synchronized with the expression of syntenic pairs across the six tissues.

### Interactions among Orthologous and Nonorthologous Pairs of BraHB Protein**s**

In the present study, orthologous and nonorthologous pairs were explored between the three subgenomes of *B*. *rapa*, as these were the evolutionary products of WGT. For identification in the *B*. *rapa* database (BRAD)^40^, we searched the pairs by “syntenic gene” among least fractionated (LF), medium fractionated (MF1), and most fractionated (MF2) subgenomes. The regulatory network was presented using Cytoscape between orthologous and nonorthologous pairs of *BraHB* genes. All the orthologous pairs of BraHB that showed more or less similarity in their relationship strength were marked in red (Supplementary Figure S4). Similarly, the 22 nonorthologous pairs that showed a close relationship were marked in green.

## Discussion

The plant kingdom contains a huge diversity of species. While they are sessile in nature, they still possess advanced characteristics of living activities. Homeobox transcription factor plays an important role in the morphogenesis of living organisms, and it begins from the very first stage of embryogenesis. To date, most research has been limited to certain plant species of *A*. *thaliana* and rice. In our research, we performed comprehensive studies of HBs in *B*. *rapa*, which includes whole genome-wide identification, chromosomal locations, phylogenetic analysis, gene duplication analysis, structural investigation, cis-elements, and interaction network. Finally, we also demonstrated the expression patterns of genes in different tissues and the response of HBs to various stress conditions.

Phylogenetic analysis revealed that *B*. *rapa* exhibits a close evolutionary relationship with model plant *A*. *thaliana*^11^. Comparative genomic studies suggest that more than 60% of the genome assemblies between *A*. *thaliana* and *B*. *rapa* are highly conserved^32,40^. Around 93% of the total predicted *B*. *rapa* gene families also appear in *A*. *thaliana*^32^. Moreover, *A*. *thaliana* genes associated with regulatory networks for environmental stimuli, such as salt, cold, light, or hormonal responses, are also highly retained in *B*. *rapa*. In Arabidopsis, approximately 100 homeobox genes were identified initially, and then classification was done depending on their domain composition and phylogenetic relationships^2^. In our study, a total of 113 BraHBs were identified and verified by using various public databases.

Most land plants have undergone polyploidization that led to whole genome duplication (WGD). This provided an opportunity for duplicated genes to diverge in different evolutionary ways. Each of these genes subsequently experienced one of three fates: subfunctionalization, neofunctionalization, or nonfunctionalization (deletion or pseudogenization)^41^. These provided opportunities for duplicated genes to gain functional diversification, resulting in more complex organisms. In addition, segmental and tandem duplications are also known to contribute mainly in duplication modes during gene family expansion^42^. In previous reports, we noticed that *B*. *rapa* not only shared three paleo-polyploidy events with *A*. *thaliana*, but also underwent a further WGT event since its divergence from *A*. *thaliana* 13 to 17 MYA^43^. For the evolution of plants, gene duplication is not the only motivation, but it has a prominent role in the expansion of the gene family along with the succession of genomic rearrangements^44^. In our study, a majority of genes (> 92%) were segmental based on duplicated analysis, whereas only five and three genes belonged to tandem and dispersed type, respectively. These results suggest that segmental duplications contributed to the expansion of the BraHB family. Meanwhile, we calculated the rate of divergence of 39 paralogous pairs. The *Ka/Ks* ratio is an indicator of the selection history of genes or gene regions. Commonly, if the value of *Ka/Ks* is lower than 1, the duplicated gene pairs may have evolved from purifying selection (also called negative selection); *Ka/Ks* = 1 means neutral selection, while *Ka/Ks* > 1 means positive selection. In this study, we further noticed that the ratio of 39 syntenic paralogous pairs was below 1.00, which may predict that they evolved from purifying selection. These results demonstrate that during subsequent evolution, the syntenic pairs of BraHB did not diverge and suggest that the purifying selection might contribute to the prevalence of the *BraHB* gene family in *B*. *rapa*. The survival of plants depends on a number of environmental cues, such as extreme high/low temperature, salinity, and the disruption of water, that could adversely affect optimal plant survival and result in low productivity. In general, both biotic and abiotic stress responses are mainly governed by hormone signaling. ABA is a phytohormone that is involved in a number of different abiotic stresses such as cold, osmotic, and drought stress^45^. The responses of different genes to multiple treatments (ABA, GA, PEG, NaCl, heat, and cold) showed significant variation in the expression profile. The results, particularly for ABA, cold stress, and other stresses, provide a valuable clue to understanding gene function and robust candidate genes to improve the abiotic stress mechanism in *B*. *rapa*. In order to understand the regulatory functions of BraHBs, overexpression techniques will shed further light on the importance of these candidate genes in response to abiotic stresses.

In conclusion, the 113 *HB* genes in Chinese cabbage are comprehensively described, including their gene structures, phylogenetic profiles, gene duplications, subcellular localizations, conserved protein motifs, and expression patterns. To date, few genes of the Chinese cabbage transcription factor superfamily have been characterized in detail (AP2/ERF, Trihelix, and bhLH)^46–48^. Therefore, this is the first comprehensive and systematic research focused on Chinese cabbage. *In silico* analysis may assist in elucidating homeobox gene family function in protein interactions, signaling pathway regulations, and defense responses under different stress conditions. Altogether, it may also provide new opportunities to discover Chinese cabbage tolerance mechanisms under stress conditions. The outcome of our bioinformatics analysis provides basic resources to examine the molecular regulation of the homeobox transcription family during development and stress conditions in Chinese cabbage. In addition, the comparative study between Chinese cabbage and other species generates valuable information to study the function of homeobox transcription factor that may result in economic, agronomic, and ecological benefit for this vegetable crop.

## Materials and Methods

### Retrieval of BraHB Sequences

The *B*. *rapa* genome sequences were downloaded from BRAD (http://brassicadb.org/brad/)^30^. The *A*. *thaliana* sequences were retrieved from The Arabidopsis Information Resource (TAIR) (http://www.arabidopsis.org/), and the sequences of rice were extracted from the Rice Genome Annotation Project (http://rice.plantbiology.msu.edu/)^49^. Based on previously reported studies^33^, their sequences (236) were used as queries at a threshold of E < 1E−4 against the *B*. *rapa* database. The hidden Markov model (HMM) profile was also used as a query in our study based on domain PF00046, downloaded from the Pfam 31.0 database (https://pfam.sanger.ac.uk/)^50^. Then we manually analyzed these potential sequences of the candidate *BraHB* genes by using the Simple Modular Architecture Research Tool (SMART) (http://smart.embl-heidelberg.de/)^51^ and National Center for Biotechnology Information (NCBI) (https://www.ncbi.nlm.nih.gov/) databases. Sequences that were found with obvious errors in their gene length and other complications were eliminated.

### Multiple Sequence Alignment and Phylogenetic Analysis

For the multiple sequence alignment of BraHB candidate genes, we performed ClustalW, using MEGA 7 software with the default options^52,53^. The phylogenetic trees were constructed using the maximum likelihood (ML) method. In order to get the reliability of resulting trees, bootstrap values of 1000 replications were performed with the Jones, Taylor, and Thornton amino acid substitution model (JTT model) using MEGA 7, while keeping the other parameters as a default. We also analyzed the nucleotide divergence among different subclasses of BraHB with the help of MEGA 7, using the Jukes–Cantor model, and its estimation was performed with 1000 bootstrap replication.

### Calculation of *Ka/Ks* and Dating of the Duplication Events

The *Ka/Ks* ratio was calculated among the paralog of BraHBs, using Clustal Omega (http://www.ebi.ac.uk/Tools/msa/clustalo/) and PAL2NAL (http://www.bork.embl.de/pal2nal/) alignment to find synonymous and nonsynonymous substitutions^54^. The divergence time among the paralog pairs was calculated with the following formula: T = Ks/2r, where Ks represents the synonymous substitutions per site and r is the rate of divergence. For dicotyledonous plants, the assumption is 1.5 synonymous substitutions per site of 10^8^ years as far as *B*. *rapa* is concerned^55^.

### Conserved Motifs, Exon-Intron Structure Analysis, and Physicochemical Parameters of BraHB Proteins

To identify conserved motif in BraHB proteins, we used Multiple EM for Motif Elicitation (MEME) software version 4.12 with the following parameters: maximum motifs 14, minimum width 12, maximum width 100; the other parameters were set as default^34^. For exon–intron structure, we used Gene Structure Display Server (GSDS) 2.0 (http://gsds.cbi.pku.edu.cn)^56^. The protein property parameters, including molecular weight (MW), isoelectronic points (pI), and grand average of hydropathy (GRAVY) values for each *BraHB* gene were calculated using the ProtParam tool (http://web.expasy.org/protparam/). The subcellular localization for BraHB proteins was conducted using the WoLF PSORT server (https://wolfpsort.hgc.jp/).

### Cis-Element Analysis and Protein Interaction Network Prediction

The promoter sequences of BraHB selected as 2000 upstream bp were retrieved from the *B*. *rapa* genome according to generic file format (GFF). Then cis-acting regulatory elements were identified for some of the specific genes using PlantCARE (http://bioinformatics.psb.ugent.be/webtools/plantcare/html/)^57^. The interaction network among orthologous and nonorthologous genes of BraHB was carried out using Cytoscape version 3.4^58^.

### Gene Chromosome Location, Gene Synteny Analysis, and Syntenic BraHB Paralogous Pair Identification

The chromosome locations of BraHBs were illustrated accordingly from top to bottom with respect to their position in genome annotation by using MapChart^59^. For synteny gene analysis, the relationships were verified between the homologues of *A*. *thaliana* and subgenomes of *B*. *rapa* (LF, MF1, and MF2) obtained from BRAD (http://brassicadb.org/brad/searchSynteny.php). The Circos program was applied to demonstrate the syntenic relationships in the chromosomes of *B*. *rapa* and *A*. *thaliana*^60^.

### Pearson Correlation Analysis

The Pearson correlation coefficient (PCC) analysis was performed using Excel 2013 to evaluate the PC values of the RNA-seq and paralogous genes that were used for qRT-PCR.

### Plant Material and Treatment

In the present study, germinated seeds of Chinese cabbage (Chiifu-401-42) were grown in plastic pots containing a mixture of soil and vermiculite (3:1). The pots were then placed in an artificial growth chamber for 5 weeks. The growth conditions were as follows: temperature was maintained at 24/16 °C, with a photoperiod of 16/8 h and relative humidity at 65–70%. The specific treatments were applied to the seedlings as follows: for heat and cold treatments, seedlings were exposed to 38 °C and 4 °C, respectively. For other stress-related treatments, plants were cultured in a nutrient medium along with a control in the following manner: (1) 100 µM ABA, (2) 100 µM GA, (3) 6000 PEG (w/v), and (4) 250 mM NaCl. Treatments were carried out in a continuous time interval of 0, 1, 6, and 12 h, respectively, with biological triplicates. Finally, all samples were frozen immediately and stored at -70 °C for further analysis.

### RNA Isolation and Expression Pattern Analysis

Total RNA was isolated from the treated frozen leaves with Trizol (Invitrogen), following the manufacturer’s instructions. RNA was reverse transcribed into cDNA using Primer Script RT reagent kit (Takara, Dalian, China) according to the manufacturer’s instructions. Specific primers were used for qRT-PCR analysis using Beacon Designer 8.1, shown in Supplementary Table S9. In order to check the specificity of the primers, we used the BLAST tool against the Brassica genome to verify them. RT-PCR was performed accordingly by following the previous report^46^. Relative fold expression was calculated with comparative Ct methods. The expression patterns of all *BraHB* genes were analyzed based on a previous study^35^. Further, gene expression levels were quantified by fragments per kilobase of transcript per million mapped reads (FPKM) values and a heatmap was generated by using OmicShare Tools (http://www.omicshare.com/).

## Electronic supplementary material

Supplementary Information

S1 Table

S2 Table

S3 Table

S4 Table

S5 Table

S6 Table

S7 Table

S8 Table

S9 Table
