# Supplementary material for: Genome-wide Identification, Classification, and Expression Pattern of Homeobox Gene Family in Brassica rapa under Various Stresses
Source: Sci Rep. 2018 Nov 2;8:16265. doi: 10.1038/s41598-018-34448-x (PMC6214979; doi:10.1038/s41598-018-34448-x)
Supplement: Supplementary file 1 — Supplementary Information [file 41598_2018_34448_MOESM1_ESM.pdf]

Genome-wide Identification, Classification, and Expression Pattern of Homeobox Gene Family in *Brassica rapa* under Various Stresses

Nadeem Khan[1], Chun-mei Hu[1, 2, *], Waleed Amjad Khan[1], Wenli Wang[1], Han Ke[1], Dong Huijie[1], Zhang Zhishuo[1] and Xilin Hou[1]

[1]State Key Laboratory of Crop Genetics and Germplasm Enhancement, Ministry of Science and Technology/College of Horticulture, Nanjing Agricultural University, Nanjing 210095, P.R.China

[2]New Rural Research Institute in Lianyungang, Nanjing Agricultural University, Nanjing, P.R. China

Corresponding Author: Chun-mei Hu

Email: jjjhcm@njau.edu.cn

Supplementary Figure Legends

Figure 1. The consensus sequence of conserved motifs of BraHBs and predicted length (amino acids) for each motif is given.

Figure (2a, 2b and 2c). Genetic distance among different sub-classes of BraHBs.

Figure 3. Showing the relationship between the three subgenomes and non-synteny ortholog in terms of the number of genes.

Figure 4. Orthologues and non-orthologous of genes of BraHB, orthologous are marked with red and non-orthologous with green. The illustration were presented using Cystoscape Software.

Fig. 5. Phylogenetic relationships of BraHBs among three species from *B. rapa*, *A. thaliana and Oryza sativa*. The phylogenetic tree was constructed by MEGA 7 using the Maximum Likelihood Method (1000 bootstrap). Gene of different species are marked with different colors.

Supplementary Table Legends

Table. 1. The basic information of *BraHB* genes identified in *Brassica rapa.*

Table. 2. Homeobox identified genes and showing the number of retention genes based on subgenomes LF, MF1 and MF2 in *Brassica rapa*.

Table. 3. Cis-element of *BraHB* genes in *Brassica rapa.*

Table. 4. The FPKM values of *BraHB* genes identified in *Brassica rapa*.

Table. 5. Syntenic Paralog pairs of BraHBs with and FPKM values.

Table. 6. The gene with outlier *Ka/Ks* values and estimated divergence time for syntenic pairs of BraHBs.

Table. 7. Relative Expression pattern of *BraHB* genes along with PCC values with respect to multiple treatments.

Table. 8. Pearson correlation coefficient of the stress-induced BraHBs whose PC are greater than 0.5

Table. 9. Sequences of the *BraHB* genes primers used for quantitative real time PCR.

	Consensus Sequences	Length (aa)
Motif 1	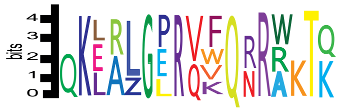	31
Motif 2	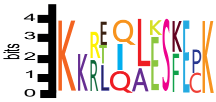	22
Motif 3	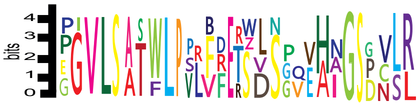	65
Motif 4	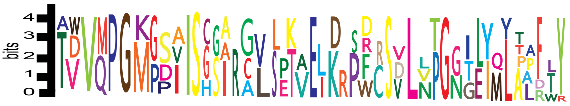	90
Motif 5	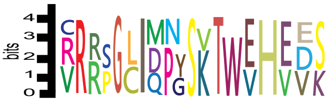	34
Motif 6	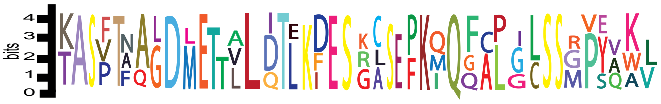	81
Motif 7	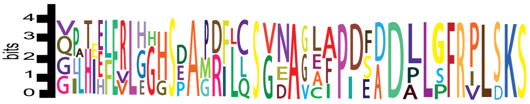	88
Motif 8	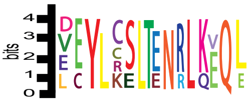	29
Motif 9	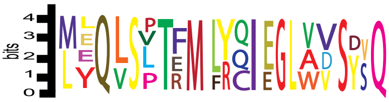	43
Motif 10	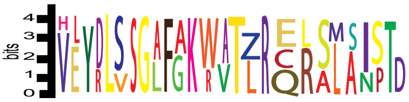	43
Motif 11	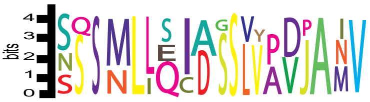	36
Motif 12	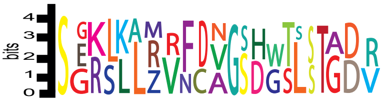	43
Motif 13	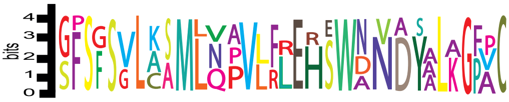	60
Motif 14	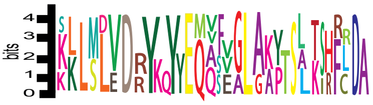	60

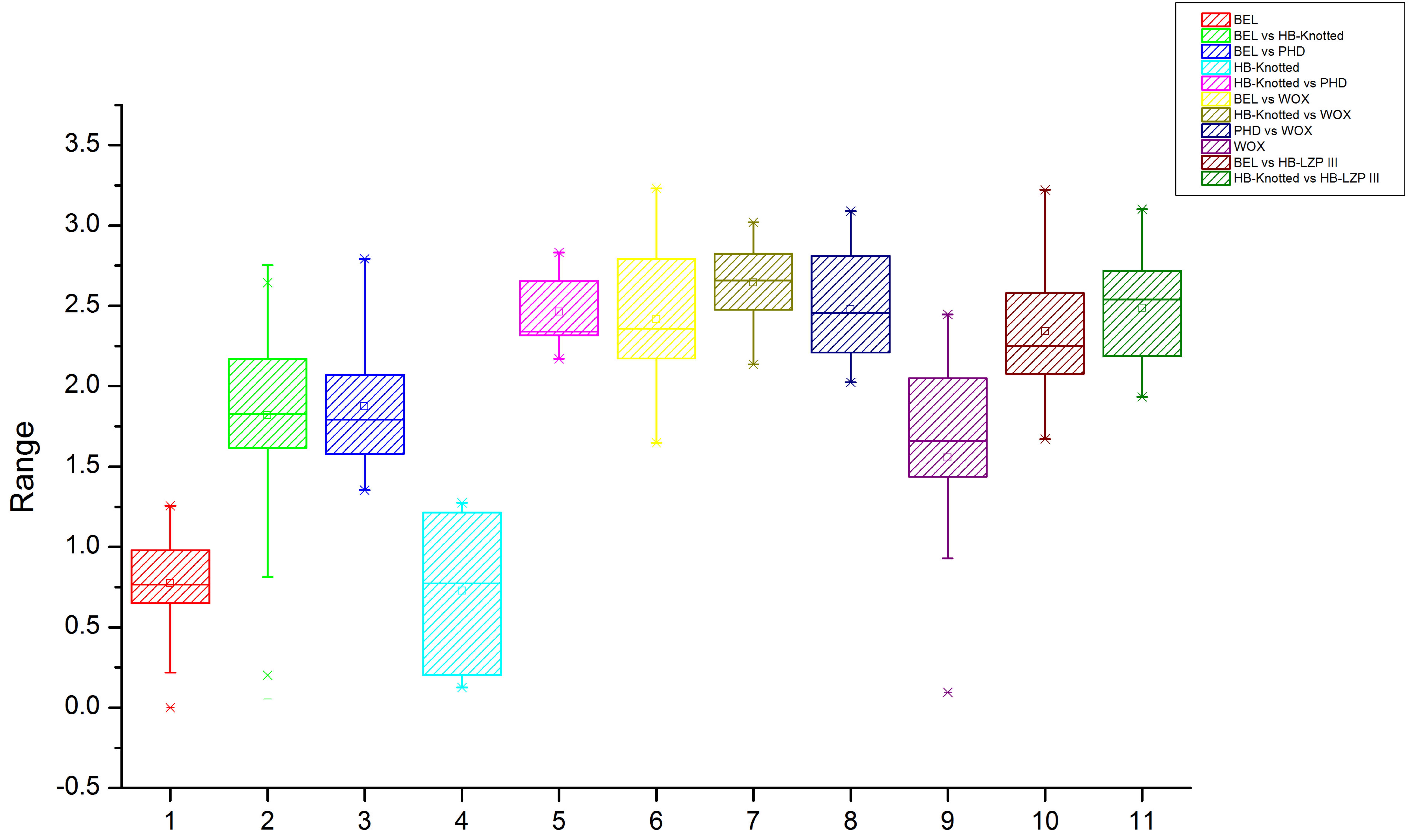
BEL
BEL vs HB-Knotted
BEL vs PHD
HB-Knotted
HB-Knotted vs PHD
BEL vs WOX
HB-Knotted vs WOX
PHD vs WOX
WOX
BEL vs HB-LZP III
HB-Knotted vs HB-LZP III
Range
3.5
3.0
2.5
2.0
1.5
1.0
0.5
0.0
-0.5
1
2
3
4
5
6
7
8
9
10
11

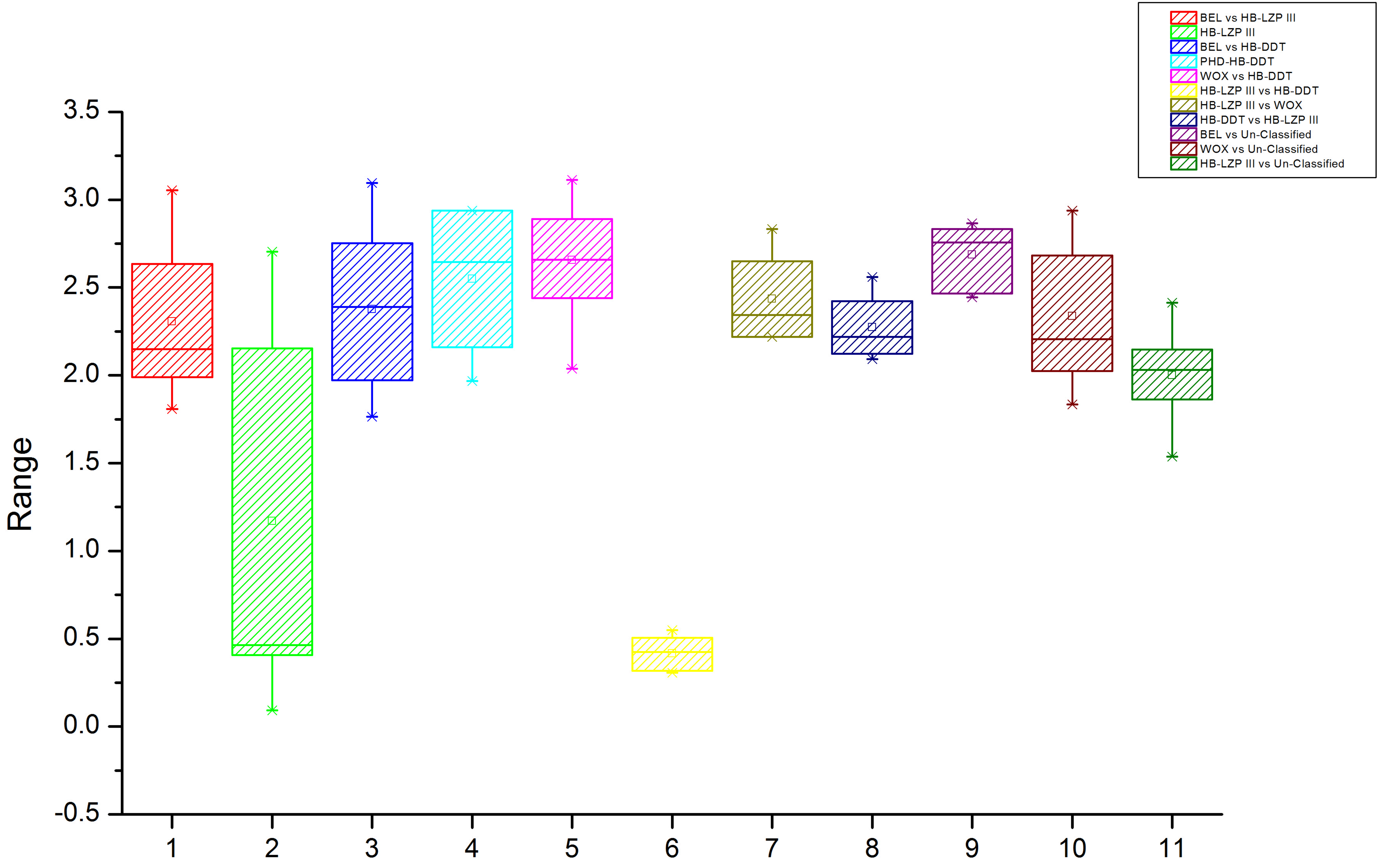
BEL vs HB-LZP III
HB-LZP III
BEL vs HB-DDT
PHD-HB-DDT
WOX vs HB-DDT
HB-LZP III vs HB-DDT
HB-LZP III vs WOX
HB-DDT vs HB-LZP III
BEL vs Un-Classified
WOX vs Un-Classified
HB-LZP III vs Un-Classified
Range
3.5
3.0
2.5
2.0
1.5
1.0
0.5
0.0
-0.5
1
2
3
4
5
6
7
8
9
10
11

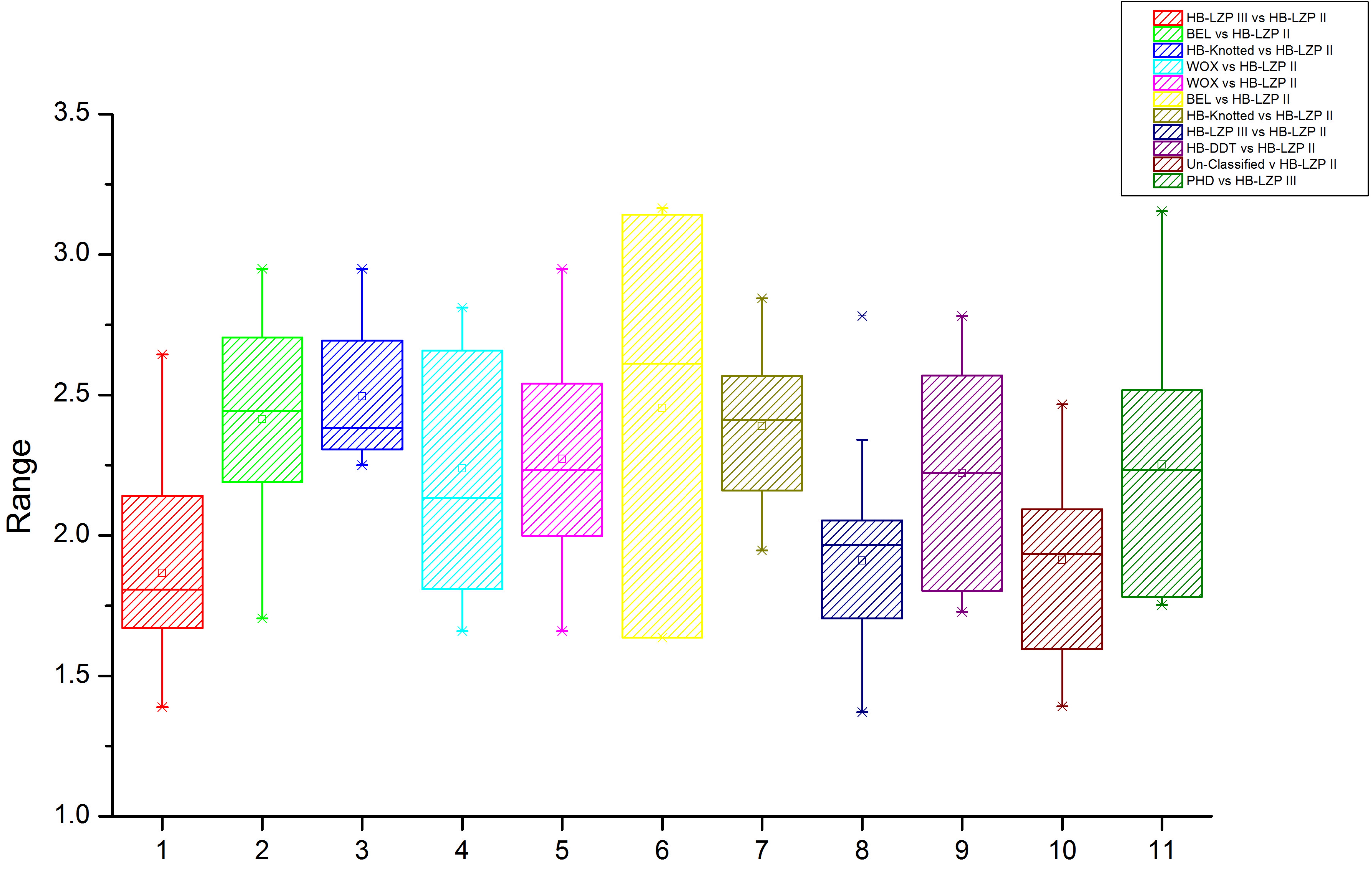

HB-LZP III vs HB-LZP II
BEL vs HB-LZP II
HB-Knotted vs HB-LZP II
WOX vs HB-LZP II
WOX vs HB-LZP II
BEL vs HB-LZP II
HB-Knotted vs HB-LZP II
HB-LZP III vs HB-LZP II
HB-DDT vs HB-LZP II
Un-Classified v HB-LZP II
PHD vs HB-LZP III
Range
3.5
3.0
2.5
2.0
1.5
1.0
1
2
3
4
5
6
7
8
9
10
11

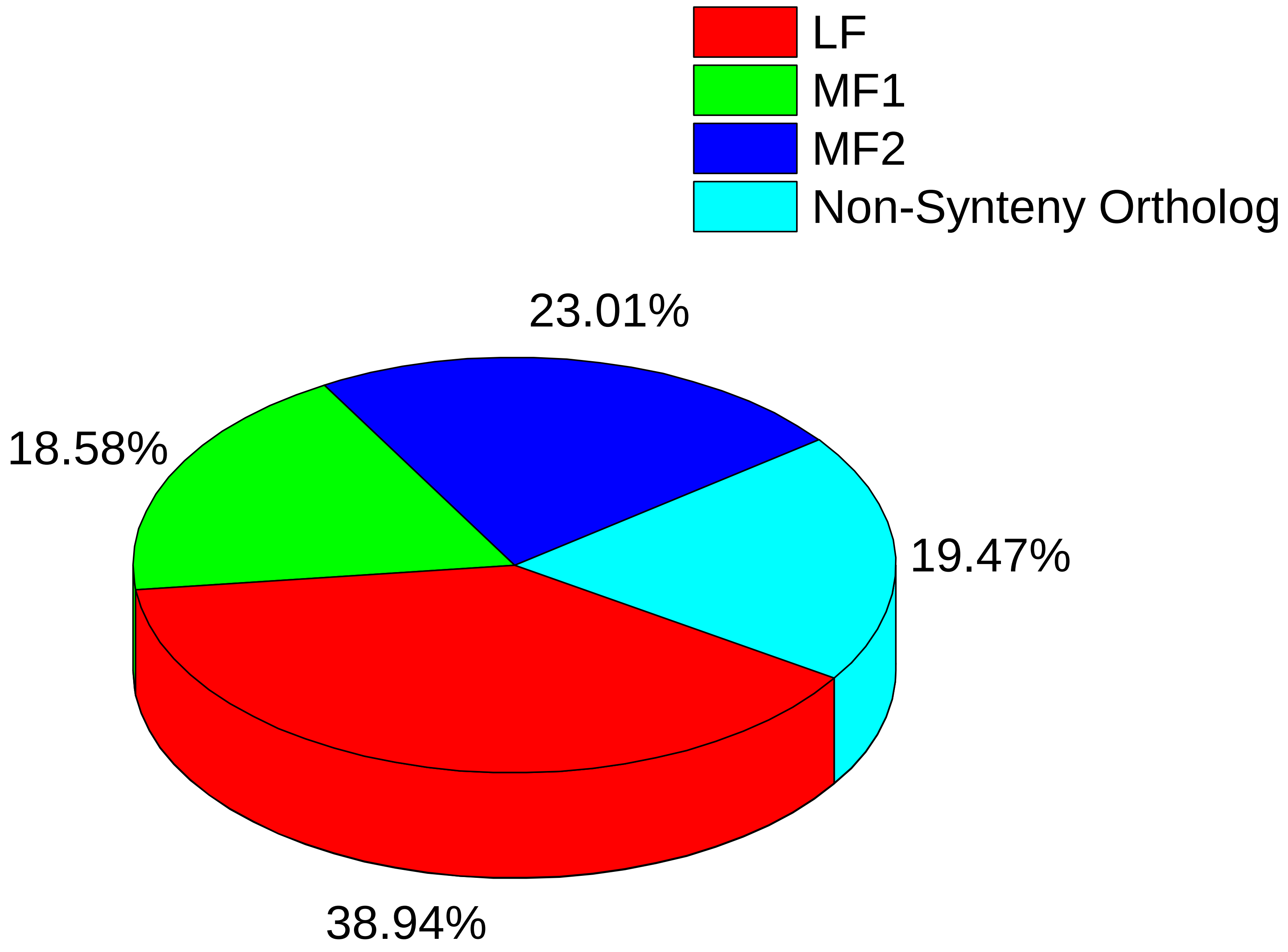

LF
MF1
MF2
Non-Synteny Ortholog
23.01%
18.58%
19.47%
38.94%

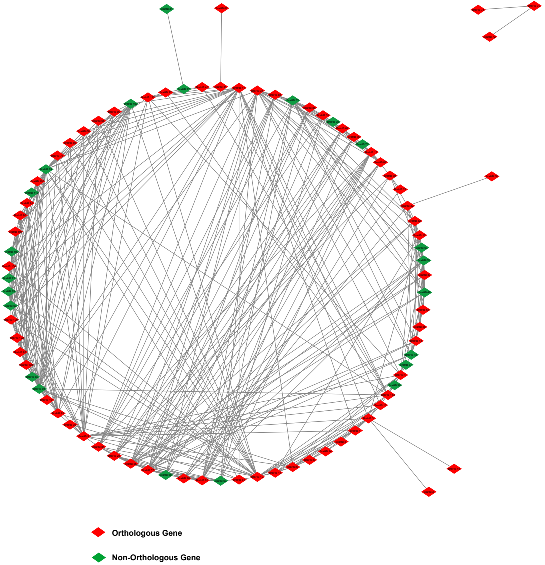
Orthologous Gene
Non-Orthologous Gene

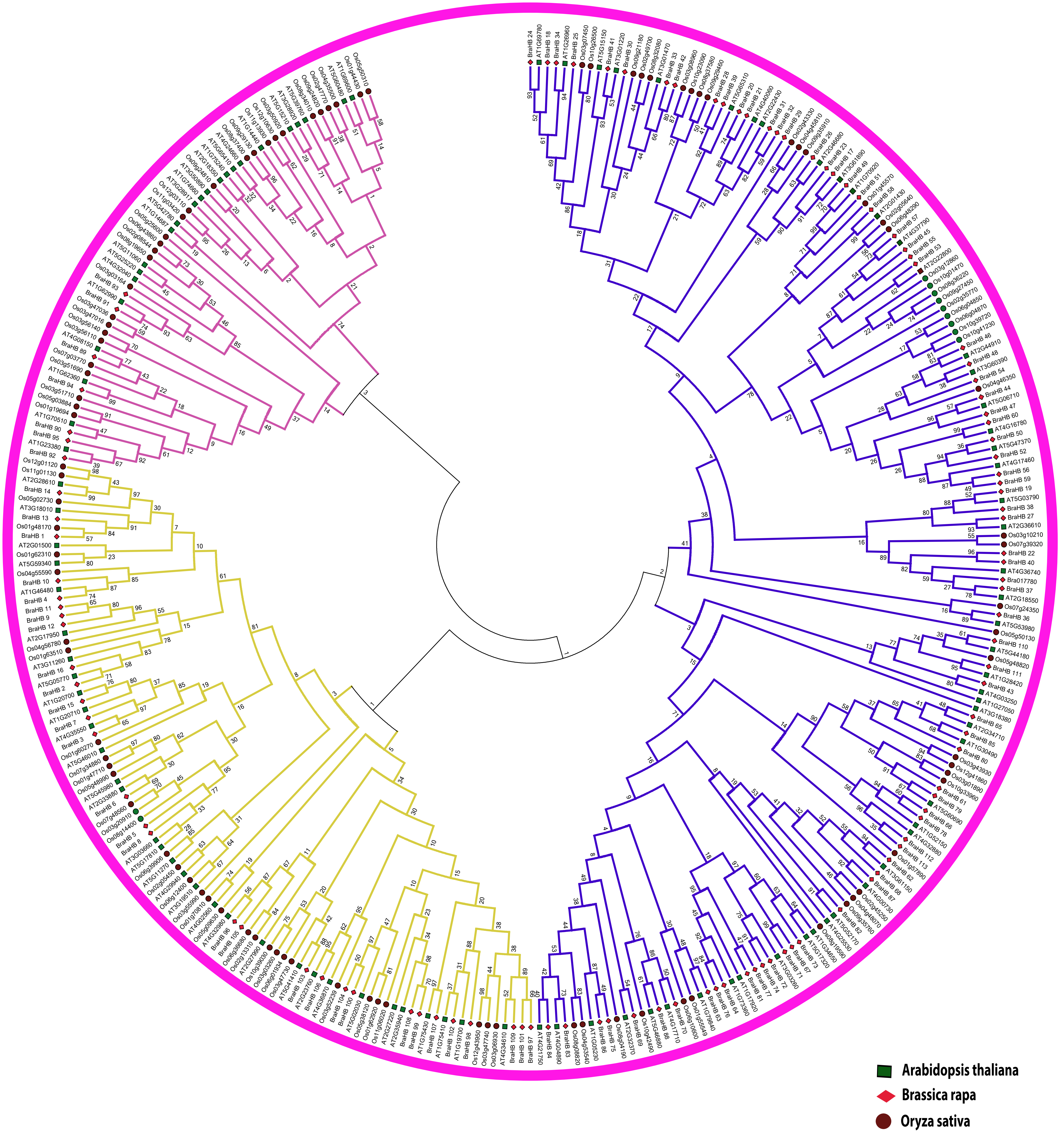
Arabidopsis thaliana
Brassica rapa
Oryza sativa
